# Letrozole and norethisterone acetate versus letrozole and triptorelin in the treatment of endometriosis related pain symptoms: a randomized controlled trial

**DOI:** 10.1186/1477-7827-9-88

**Published:** 2011-06-21

**Authors:** Simone Ferrero, Pier L Venturini, David J Gillott, Valentino Remorgida

**Affiliations:** 1 Department of Obstetrics and Gynaecology, San Martino Hospital and University of Genoa, Italy; 2 St. Bartholomew's School of Medicine & Dentistry, Queen Mary University of London, London, UK

## Abstract

**Background:**

When aromatase inhibitors are used to treat premenopausal women with endometriosis, additional drugs should be used to effectively down-regulate gonadal estrogen biosynthesis. This randomized prospective open-label study compared the efficacy in treating pain symptoms and the tolerability of letrozole combined with either norethisterone acetate or triptorelin.

**Methods:**

Women with pain symptoms caused by rectovaginal endometriosis were treated with letrozole (2.5 mg/day) and were randomized to also receive either oral norethisterone acetate (2.5 mg/day; group N) or intramuscular injection of triptorelin (11.25 mg every 3 months; group T). The scheduled length of treatment was 6 months. A visual analogue scale and a multidimensional categorical rating scale were used to assess the severity of pain symptoms. The volume of the endometriotic nodules was estimated by ultrasonography using virtual organ computer-aided analysis. Adverse effects of treatment were recorded.

**Results:**

A total of 35 women were randomized between the two treatment protocols. Significantly more patients in group N rated their treatment as satisfactory or very satisfactory (64.7%) as compared to group T (22.2%; p = 0.028). The intensity of both non-menstrual pelvic pain and deep dyspareunia significantly decreased during treatment in both study groups, though no statistically meaningful difference between the two groups was apparent. Reduction in the volume of endometriotic nodules was significantly greater in group T than in group N. Interruption of treatment due to adverse effects significantly differed between the groups, with 8 women in group T (44.4%) and 1 woman in group N (5.9%) interrupting treatment (p = 0.018). Similarly, 14 women included in group T (77.8%) and 6 women included in group N (35.3%) experienced adverse effects of treatment (p = 0.018). During treatment, mineral bone density significantly decreased in group T but not in group N.

**Conclusions:**

Aromatase inhibitors reduce the intensity of endometriosis-related pain symptoms. Combining letrozole with oral norethisterone acetate was associated with a lower incidence of adverse effects and a lower discontinuation rate than combining letrozole with triptorelin.

## Background

Over the last 10 years, several studies showed that the administration of aromatase inhibitors significantly reduces the severity of pain symptoms caused by endometriosis [1]. In premenopausal women, aromatase inhibitors decrease the concentration of circulating estrogens and cause an increase in FSH secretion leading to a stimulatory effect on the growth of ovarian follicles [2]. In line with this, it has been shown that the daily oral administration of letrozole and desogestrel in women with rectovaginal endometriosis results in the development of functional ovarian cysts [3]. Similarly, functional ovarian cysts developed in over 50% of patients with symptomatic uterine leiomyomas treated with letrozole monotherapy for three months [4] and in 24% of women receiving letrozole for two months after laparoscopic treatment of endometriosis [5]. Therefore, when aromatase inhibitors are administered to premenopausal women, additional drugs should be used to effectively down-regulate the ovaries and gonadal estrogen biosynthesis [6].

Previous studies in women with endometriosis combined aromatase inhibitors (letrozole or anastrozole) with combined oral contraceptive pills [7], norethisterone acetate [8-12] or gonadotropin-releasing hormone analogues [13,14]. However, there are currently no published studies comparing pain symptoms and adverse effects when gonadotropin-releasing hormone analogue and progestin are administered in combination with aromatase inhibitors.

Given this background, the current study investigated whether the administration of progestin or gonadotropin-releasing hormone analogue in combination with letrozole has different efficacy and tolerability in women with rectovaginal endometriosis.

## Methods

This prospective, randomized, open-label trial compared the efficacy of letrozole combined with either norethisterone acetate or triptorelin in the treatment of pain symptoms caused by rectovaginal endometriosis. The study was performed in an academic centre for the diagnosis and treatment of endometriosis. The primary end point of the study was to compare the changes in pain symptoms during the 6-month treatment with the two study protocols. The secondary objective of the study was to evaluate the incidence of adverse effects. The tertiary objective of the study was to evaluate the changes in the volume of the rectovaginal nodules during treatment.

The local Institutional Review Board approved the study protocol. The patients enrolled in the study signed a written informed consent.

### Study population

Women who participated had previously undergone laparoscopy or laparotomy for symptomatic endometriosis in other hospitals but deep endometriotic lesions were not excised; however, the presence of endometriosis was histologically diagnosed. These patients had recurrent or persistent pain symptoms after surgery. Patients included in the study had pain symptoms of more than 12-months duration and wished to avoid further surgery. Only premenopausal women were included in the study.

The diagnosis of rectovaginal endometriosis was based on vaginal and rectal examinations and confirmed by rectal water contrast transvaginal ultrasonography [15-17]. Patients with gastrointestinal complains suggestive of bowel endometriosis underwent multidetector computerized tomography enteroclysis [18-20]. Kidney and urinary tract evaluations were always performed.

The exclusion criteria for the study were: uropathy or endometriotic nodules determining bowel stenosis; ovarian endometrioma of diameter > 3 cm; therapies for endometriosis other than nonsteroidal anti-inflammatory drugs in the three months before inclusion in the study (six months for GnRH analogues); previous use of aromatase inhibitors; unwillingness to tolerate menstrual changes; undiagnosed vaginal bleeding; osteopenia or osteoporosis; current or past history of seizure disorders; pulmonary, cardiac, hepatic, or renal diseases; thromboembolic or cerebrovascular events; pregnancy; psychiatric disturbances; and history of drug or alcohol abuse.

### Study protocol and randomization

All study patients received letrozole (2.5 mg/day, Femara; Novartis Farma, Varese, Italy), elemental calcium (1000 mg/day), and vitamin D3 (880 IU/day, Cacit-Vitamina D3; Procter & Gamble, Rome, Italy; group L). In addition, they were randomized to receive either oral norethisterone acetate (2.5 mg/day, Primolut-Nor; Schering, Milan, Italy; group N) or depot intramuscular injection of the 3-month formulation of triptorelin (11.25 mg; Decapeptyl, Ipsen Pharma, Milan, Italy; group T). The scheduled length of treatment was 6 months.

Randomization was performed 1:1 by using a computer-generated randomization list prepared by an independent statistician not involved in the rest of the investigation. Based on the list, sequentially numbered sealed opaque envelope containing cards with group assignment were prepared. These sealed envelopes marked with the patients' sequential numbers were kept at the endometriosis clinic. When a patient was enrolled and written informed consent obtained, the envelope with the lowest number was opened and allocation to treatment was assigned.

Subjects were allowed to take nonsteroidal anti-inflammatory drugs when needed (naproxen sodium, 550 mg tablet, Synflex Forte 550, Recordati Industria Chimica e Farmaceutica, Milan, Italy); however, they were asked to record the number of tablets used each month during treatment.

Complete blood count, serum electrolytes, kidney and liver function tests, along with lipids were performed before the onset of therapy, every two months during treatment, and at the completion of treatment. A bone densitometry determination of the hip and lumbar spine (by dual-energy X-ray absorptiometry or DEXA scan) was performed within one month before the onset of the study and was repeated within one month after completion of the treatment.

### Evaluation of symptoms

Each patient was asked to complete a questionnaire on the presence and severity of dysmenorrhea, nonmenstrual pelvic pain, and deep dyspareunia. The severity of pain symptoms was measured using a 10-cm visual analogue scale (VAS) as previously described [10]. A score of 0.1 to 5.0 was considered mild pain, 5.1 to 8.0 moderate pain, and 8.1 to 10.0 severe pain. Patients enrolled in the study had at least one moderate or severe symptom. In addition, patients were asked to complete a questionnaire investigating the presence and severity of dysmenorrhea, deep dyspareunia, and nonmenstrual pelvic pain graded with a 0-to 3-point multidimensional categorical rating scale modified from the one devised by Biberoglu and Behrman [21] and previously described by other authors [22]. This scale defines dysmenorrhea according to loss of work efficiency and need for bed rest (absence of pain, 0; some loss of work efficiency, mild, 1; in bed part of 1 day, occasional loss of work, moderate, 2; in bed for 1 or more days, incapacitation, severe, 3); nonmenstrual pelvic pain according to various degrees of discomfort and use of analgesics (absence of pain, 0; occasional pelvic discomfort, mild, 1; noticeable discomfort for most of the cycle, moderate, 2; pain persisting during the cycle or requiring strong analgesics, severe, 3); and deep dyspareunia according to limitation of sexual activity (no discomfort, 0; tolerated discomfort, mild, 1; intercourse painful to the point of interruption, moderate, 2; intercourse avoided because of pain, severe, 3).

Severity of symptoms was evaluated before starting the treatment and after 3 and 6 months of treatment.

After the completion of treatment or at the time of interruption of treatment, the women rated the overall degree of satisfaction with their treatment by answering to the following question: "Taking into consideration the variations in pain symptoms, in overall well-being and quality of life, as well as the adverse effects experienced, if any, how would you define the level of satisfaction with your treatment?" as described previously [23,10]. Answers were based on a 5-point Likert scale (very satisfied, satisfied, uncertain, dissatisfied, very dissatisfied). Adverse effects experienced during the 6-month treatment were recorded during monthly consultations.

### Evaluation of the volume of rectovaginal nodules

The volume of the endometriotic nodules was estimated by ultrasonography before commencement and after completion of 6 months hormonal therapy. Ultrasound examinations were performed using a Voluson i ultrasound machine (GE Healthcare, Milwaukee, WI, USA) connected to a transvaginal transducer. The volume of the rectovaginal endometriotic nodules was estimated by virtual organ computer-aided analysis (VOCAL™, GE Healthcare, Milwaukee, WI, USA) [24]. The VOCAL™ technique was used to obtain a sequence of 20 sections of each endometriotic nodule around a fixed axis, from the proximal to the distal part of the nodule, each after 9° rotation from the previous section, which represents the best compromise between reliability, validity and time to define the volume [25]. The contour of each nodule was drawn manually by using the roller ball cursor of the 3D ultrasound machine to obtain a 3D volume measurement. Each measurement was performed off-line after scanning by a single trained operator who was not aware of the type of hormonal therapy administered to the patients. The time required to perform these measurements ranged from 10 to 15 minutes.

### Power analysis

In calculating the sample size required for this randomized study, it was considered that a previous study including women with rectovaginal endometriosis reported that 56% of the patients were satisfied after 6-month treatment with norethisterone acetate and letrozole [10]. A difference of 30% in the satisfaction rate between the two study groups was considered clinically relevant. To have an 80% chance of detecting such a difference at an overall statistical significance level of 5%, 35 patients per group were required. Allowing for dropouts, the aim was to recruit a total of about 80 women. The study was ended pre-term based on the results of an interim analysis.

### Statistical analysis

The baseline characteristics of the study population were compared by using the student t test, χ^2 ^test or Fisher's exact test as appropriate. The student t test was used to compare the intensity of pain symptoms measured on the VAS scale between the two study groups. The Mann-Whitney rank sum test was used to compare the intensity of pain symptoms measured on the multidimensional categorical rating scale between the two study groups. The paired t-test and the Wilcoxon signed rank test were used to compare the intensity of pain symptoms before and after treatment. P < 0.05 was considered statistically significant. Data were analyzed using the Sigma Stat software version 3.5 and the SPSS software version 13.0 (SPSS Science, Chicago, IL, USA).

## Results

**Figure 1:**
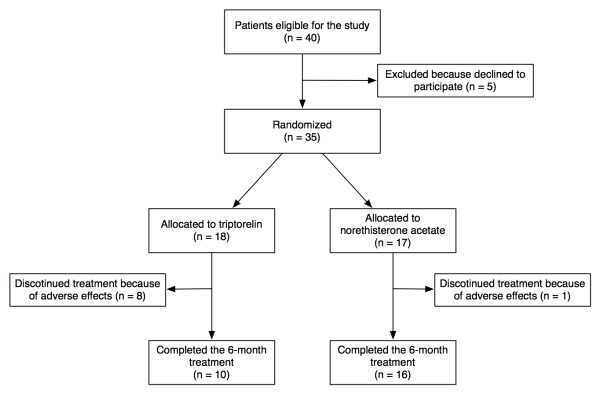
**Flow chart showing recruitment and women's progress through the study**.

The diagrammatic flow of the participants is given in Figure 1. Out of 40 women approached for the study, 35 patients (87.5%) gave their consent and were randomized to receive one of the treatments. At the time of the interim analysis, 18 were allocated to group T and 17 women were allocated to group N.

The mean ( ± SD) age was similar in group T (35.0 ± 3.6 years) and in group N (35.2 ± 4.0; p = 0.857).

Eight women in group T (44.4%) and 1 woman in group N interrupted the treatment because of adverse effects (5.9%) (p = 0.018). In group T, the 8 patients interrupted the treatment after a mean ( ± SD) time of 3.9 ( ± 1.0) months; the woman in group N interrupted the treatment after 4 months.

After the completion of treatment or at the time of interruption of treatment, in group T, 2 women (11.1%) were very dissatisfied, 10 women (55.6) were dissatisfied, 2 women (11.1%) were uncertain, 3 (16.7%) women were satisfied and 1 woman (5.6%) was very satisfied. In group N, 1 woman (5.9%) was very dissatisfied, 4 women (23.5%) were dissatisfied, 1 (5.9%) woman was uncertain, 8 women (47.1%) were satisfied and 3 (17.6%) women were very satisfied. Therefore, 4 women (22.2%) were satisfied or very satisfied in group T and 11 women (64.7%) were satisfied or very satisfied in group N (p = 0.028).

Table 1 shows the intensity of pain symptoms at baseline and during treatment. The baseline intensity of the symptoms was similar in the two study groups both on the VAS scale and on the multidimensional categorical rating scale.

**Table 1:** Intensity of pain symptoms at baseline and during treatment

	VAS scale			Multidimensional categorical rating scale		
	group T (n = 18)	group N (n = 17)	p	group T (n = 18)	group N (n = 17)	p
**Dysmenorrhea**						
- baseline	8.7 ± 1.1 (n = 18)	8.6 ± 1.3 (n = 16)	0.881	3 (2-3) (n = 18)	3 (0-3) (n = 17)	0.890
**Nonmenstrual pelvic pain**						
- baseline	6.1 ± 1.4 (n = 16)	6.0 ± 1.4 (n = 14)	0.783	2 (0-3) (n = 18)	2 (0-3) (n = 17)	0.957
- 3 months of treatment	3.2 ± 1.3 (n = 15)	3.3 ± 1.5 (n = 14)	0.782	1 (0-2) (n = 17)	1 (0-2) (n = 17)	0.818
- 6 months of treatment	1.2 ± 1.3 (n = 9)	2.0 ± 1.8 (n = 14)	0.286	0 (0-2) (n = 10)	1 (0-2) (n = 16)	0.171
p value: 3 month of treatment vs baseline	< 0.001	< 0.001		< 0.001	< 0.001	
p value: 6 month of treatment vs baseline	< 0.001	< 0.001		0.004	< 0.001	
p value: 6 month of treatment vs 3 month	0.001	< 0.001		0.063	0.156	
**Deep dyspareunia**^a^						
- baseline	6.4 ± 1.9 (n = 12)	6.6 ± 2.1 (n = 14)	0.801	2 (0-3) (n = 14)	2 (0-3) (n = 15)	0.501
- 3 months of treatment	3.4 ± 1.2 (n = 11)	3.6 ± 1.5 (n = 14)	0.635	1 (0-2) (n = 13)	1 (0-2) (n = 15)	0.853
- 6 months of treatment	2.0 ± 0.9 (n = 4)	2.2 ± 1.4 (n = 13)	0.727	1 (0-1) (n = 6)	1 (0-2) (n = 14)	0.406
p value: 3 month of treatment vs baseline	< 0.001	< 0.001		0.031	< 0.001	
p value: 6 month of treatment vs baseline	0.022	< 0.001		0.042	< 0.001	
p value: 6 month of treatment vs 3 month of treatment	0.088	< 0.001		0.076	0.125	

^a^14 women included in group T and 15 women included in group N were sexually active at the time of the study

The intensity of each pain symptom was measured only for patients suffering the symptom; the multidimensional categorical rating scale was calculated for all the patients because this scale includes the 0 for patients not suffering the symptom

The intensity of both nonmenstrual pelvic pain and deep dyspareunia were significantly lower at 3-and 6-month when compared with baseline values in both study groups. The intensity of pain symptoms was similar in group T and in group N at both 3-and 6-month treatment in both scales.

When the intensity of pain symptoms at 6-month was compared with the intensity of pain symptoms at 3-month, a significant decrease of nonmenstrual pelvic pain measured on the VAS scale was observed in group T and group N. The intensity of deep dyspareunia decreased significantly in group N; there was a trend for a lower intensity of deep dyspareunia at 6-month compared with 3-month in group T, but the difference did not reach statistical significance, possibly due to the limited number of patients who completed the 6-month treatment in this group. In both study groups, there was no significant difference in the multidimensional categorical rating scale scores between 3-and 6-month assessments.

At baseline, the volume of the rectovaginal endometriotic nodules was similar in group T (mean ± SD, 3.2 ± 0.9 cm^3^) and in group N (3.4 ± 1.0 cm^3^; p = 0.689). After 6 months of treatment, there was a significant reduction in the volume of the endometriotic nodules in both study groups (group T, p = 0.001; group N, p < 0.001). The mean ( ± SD) percentage reduction in the volume of the endometriotic nodules was significantly higher in group T (16.1 ± 10.0%) than in group N (10.2 ± 6.3%; p = 0.048).

The mean ( ± SD) number of naproxen sodium tablets used per patient each month was 6.7 ( ± 4.5) in group T and 6.5 ( ± 5.0) in group N (p = 0.904). When compared with baseline values, the mean ( ± SD) number of naproxen sodium tablets used per patient each month after 3 months of treatment significantly decreased both in group T (2.6 ± 2.2; p < 0.001) and in group N (2.6 ± 2.8; p < 0.001). After 6 months of treatment, the number of monthly naproxen sodium tablets used per patient was further decreased in group N (1.4 ± 1.3) when compared with 3-month treatment (p = 0.042). After 6-month treatment, in group T, there was a trend for the number of naproxen sodium tablets used per patient each month to be lower (1.8 ± 1.2) than at 3-month treatment but the difference did not reach statistical significance (p = 0.070).

14 women included in group T (77.8%) and 6 women included in group N (35.3%) experienced adverse effects of treatment (p = 0.018). Table 2 shows the adverse effects of treatment experienced by patients included in the two study groups; arthralgia was significantly more frequent in group T than in group N. In group T, the main reasons for interruption of treatment were arthralgia in 5 women, hot flushes and hair loss in 2 women, decreased libido in 2 women, arthralgia and hot flushes in 1 woman, arthralgia and myalgia in 1 woman, hot flushes and depression in 1 woman, myalgia in 1 woman and depression in 1 woman. In group N, the main reasons for interruption of treatment were weight gain in 2 women, arthralgia in 1 woman, breakthrough bleeding in 1 woman, depression in 1 woman and decreased libido in 1 woman.

**Table 2:** Adverse effects of treatment experienced by patients included in the study

Adverse effect	group T (n = 18)	group N (n = 17)	p
Arthralgia	7 (38.9%)	1 (5.9%)	0.041
Myalgia	3 (16.7%)	1 (5.9%)	0.603
Persistent breakthrough bleeding	0 (0.0%)	2 (11.8%)	0.229
Depression	4 (22.2%)	1 (5.9%)	0.338
Insomnia	3 (16.7%)	0 (0.0%)	0.229
Decreased libido	4 (22.2%)	2 (11.8%)	0.658
Vaginal dryness	3 (16.7%)	0 (0.0%)	0.229
Hot flushes	4 (22.2%)	0 (0.0%)	0.104
Hair loss	2 (11.1%)	0 (0.0%)	0.486
Headache	2 (11.1%)	0 (0.0%)	0.486
Weight gain	1 (5.6%)	2 (11.8%)	0.603
At least one adverse effect	14 (77.8%)	6 (35.3%)	0.018

There were no adverse effects on blood count, liver function, renal function, and lipid profile (data not shown).

At the completion of the 6-month treatment, DEXA scans showed that patients included in group T had a significant decrease in the mineral bone density both in the lumbar spine (p = 0.019) and in the hip (p = 0.002). In group N, DEXA scans showed no significant change in the mineral bone density both in the lumbar spine (p = 0.192) and in the hip (p = 0.221). No woman fell into the category of osteopenia at the conclusion of the treatment.

## Discussion

Hormonal therapies are not curative of endometriosis, therefore, they should be chronically administered to women with endometriosis [26]. Viewed from this perspective, the incidence of adverse effects is particularly relevant because they may affect compliance to therapy. This randomized prospective study compared two different therapeutic regimens, demonstrating that co-treatment with progestin is more accepted by the patients that co-treatment with gonadotropin-releasing hormone analogue. In fact, the incidence of adverse effect is significantly higher when letrozole is combined with triptorelin than when it is combined with norethisterone acetate. In fact, 77.8% of women included in group T and 35.3% of those included in group N had at least one adverse effect. In line with this, the percentage of patients who interrupted the treatment was significantly higher in group T than in group N (44.4% versus 5.9%). Because of these reasons, the study was terminated before it reached the full enrolment of 80 subjects.

The risk of adverse effects during treatment with aromatase inhibitors is related to the length of treatment. A short-term administration of aromatase inhibitors (two or three months) may not cause significant adverse effects; in the current study only two women (5.7%) interrupted the treatment before the fourth month of therapy because of adverse effects. This observation is consistent with a recent study which reported no significant adverse effect of administering letrozole for two months after laparoscopic treatment of endometriosis [25]. However, several previous studies showed that a longer administration of aromatase inhibitors (six months) might be associated with several adverse effects [3,7-12]. In contrast with these observations, a prospective randomized trial comparing the postsurgical administration of goserelin plus anastrozole to goserelin alone for 6 months did not describe the occurrence of typical aromatase inhibitor related adverse effects (such as arthralgia and myalgia) [13]. It is possible, that the patients included in our study were more active than those receiving aromatase inhibitors in the postoperative period and exhibited lower tolerance of adverse effects that may impair their daily activities. However, in another study, reported in abstract form, 90 women with pain symptoms relapsing after surgical and medical treatments were randomized to receive either anastrozole and goserelin or goserelin alone for six months [14]. After a follow-up of at least two years, patients receiving the double drug regimen showed a significantly lower relapse of pain than those receiving goserelin alone; however, no adverse effect of aromatase inhibitors was reported and there was no evidence of higher discontinuation rates in patients receiving the double-drug regimen. The reasons of these differences in the incidence of adverse effects and discontinuation rate remain unclear. It is possible that the monthly consultations performed in the current study increased the reporting of adverse effects.

This study confirms the previous finding that combined administration of aromatase inhibitors and gonadotropin-releasing hormone analogues for 6 months is associated with a reduction in bone mineral density [13]. However, at the completion of treatment, no significant change in mineral bone density was observed in women included in group N confirming our own previous observations [9,11] and by other authors [8]. This finding might be explained by evidence that norethisterone acetate may have a positive effect on bone metabolism [27]. In fact, a small fraction of norethisterone acetate (between 0.20 and 0.33%) is converted to ethinyl estradiol [28].

Although the current study was ended preterm, our results confirm that aromatase inhibitors combined with ovarian suppressive agents significantly reduce the severity of endometriosis-related pain symptoms [1]. No significant difference was observed in the reduction in pain symptoms between patients receiving triptorelin and those receiving norethisterone acetate. However, because of the small number of patients included in the study, no definitive conclusion can be drawn regarding the effectiveness of the two treatments in reducing the severity of pain symptoms in women with rectovaginal endometriosis.

Previous studies examined the changes in the volume of rectovaginal endometriotic nodules during hormonal therapy demonstrating that the administration of progestin [23], oral contraceptive pill [23], vaginal danazol [29], gonadotropin-releasing hormone analogue [30] and the levonorgestrel intrauterine device [31] for 6 to 12 months significantly reduces the size of rectovaginal endometriotic nodules. The current study combining letrozole with either progestin or gonadotropin-releasing hormone analogue confirmed that hormonal therapy significantly decreases the volume of rectovaginal endometriotic nodules. Interestingly, it was observed that the patients receiving triptorelin had a significantly higher percentage reduction in the volume of the nodules than those receiving norethisterone acetate.

## Conclusions

In conclusion, the current study confirms the efficacy of aromatase inhibitors in treating endometriosis-related pain symptoms. Because of the small number of patients included in the study, a definitive conclusion cannot be drawn on the superior efficacy of one treatment over the other in relieving pain symptoms caused by rectovaginal endometriosis. This study shows, for the first time, that co-treatment with progestin is more accepted by the patients. In fact, combining the aromatase inhibitor with gonadotropin-releasing hormone analogues may be associated with a higher incidence of adverse effects and, consequently, a higher discontinuation rate than combining aromatase inhibitors with progestins. Based on these finding, progestins should be the first line choice to down-regulate the ovaries in premenopausal women receiving aromatase inhibitors for the treatment of endometriosis. Future studies might investigate whether combined oral contraceptives have efficacy and tolerability similar to progestins when used to suppress ovarian activity during treatment with aromatase inhibitors.

## Competing interests

The authors declare that they have no competing interests.

## Authors' contributions

SF and VR designed the study. DJG performed the power analysis. SF, PLV and VR recruited the patients in the study. PLV and VR prescribed the treatment and evaluated the symptoms. SF measured by ultrasonography the volume of the rectovaginal endometriotic nodules. DJG performed the statistical analysis and interpreted the findings. SF wrote the preliminary draft of the manuscript, which was reviewed by PLV, DJG and VR. DJG performed the revision of the manuscript. All authors read and approved the final manuscript.
